# Impact of copper and cobalt-based metal-organic framework materials on the performance and stability of hole-transfer layer (HTL)-free perovskite solar cells and carbon-based

**DOI:** 10.1038/s41598-024-62977-1

**Published:** 2024-06-04

**Authors:** Faezeh Arjmand, Zohreh Rashidi Ranjbar

**Affiliations:** Department of Chemistry, Shahid Bahonar University of Kerman, Kerman, 76169-133 Iran

**Keywords:** Perovskite solar cell, Metal-organic frameworks, Interlayer, Efficiency, Chemistry, Nanoscience and technology

## Abstract

This article investigates the impact of metal-organic frameworks (MOFs) on the performance and stability of perovskite solar cells (PSCs), specifically focusing on the type of metal and the morphology of the MOF. Two types of MOFs, copper-benzene-1,3,5-tricarboxylate (Cu-BTC MOF) with spherical morphology and cobalt-benzene-1,3,5-tricarboxylate (Co-BTC MOF) with rod morphology, are synthesized and spin-coated on TiO_2_ substrates to form FTO/TiO_2_/MOF/CH_3_NH_3_PbI_3_/C-paste PSCs. The morphology and size of the MOFs are characterized by scanning electron microscopy (SEM), and the crystallinity and residual PbI_2_ of the perovskite films are analyzed by X-ray diffraction (XRD). The results show that the Co-BTC MOF PSC exhibits the highest power conversion efficiency (PCE) of 10.4% and the best stability, retaining 82% of its initial PCE after 264 h of storage in ambient air. The improved performance and stability are attributed to the enhanced crystallinity and reduced residual PbI_2_ of the perovskite film after Co-BTC MOF modification. The paper showcases the immense potential of MOF-based interlayers to revolutionize PSC technology, offering a path toward next-generation solar cells with enhanced performance and longevity.

## Introduction

Perovskite solar cells, based on organic–inorganic metal halide perovskites, have become highly desirable semiconductors for efficient light harvesting in photovoltaic (PV) applications because of their exceptional optoelectronic properties, which include a high tolerance for defects, a long diffusion length of charges, and a high absorption coefficient for light^1^. The field of perovskite solar cells has experienced remarkable progress over the past decade since their initial discovery in 2009^1^. The power conversion efficiency of PSCs has soared from an initial value of 3.8% to a recently certified efficiency of 26.1%^1–10^. This achievement not only surpasses the performance of other thin-film PV technologies but also rivals that of crystalline silicon solar cells, which are considered the gold standard in the solar industry. Despite the notable accomplishments in PV performance, the limited durability of PSCs continues to hinder their widespread commercialization. As a result, substantial efforts have been made to improve PSCs’ long-term stability through the use of different stabilization techniques. These tactics include device structural optimization, compositional engineering, surface and interfacial passivation, and the application of cutting-edge encapsulation methods^11^. The remarkable performance of a PSC is closely linked to the inherent characteristics of each functional layer. The absorption layer composed of perovskite material needs to possess high light absorption capability and minimal carrier recombination rates to effectively capture photons and transport charge carriers. To enhance the photo-harvesting and charge-separation efficiencies, it is crucial to have a well-crystallized perovskite layer with a low density of traps^12^. This ensures efficient collection of photons and promotes effective charge transport, resulting in improved overall performance of the PSC. Metal–organic frameworks, known as a type of porous material, offer a remarkable level of structural flexibility and diverse chemical functionality due to their customizable building blocks. MOFs exhibit extraordinary chemical resistance to polar solvents and water molecules due to the tight coordination between their unique organic linkers and metal ion nodes. Because of this special quality, MOFs can function as functional interlayers or mesoporous frameworks, which significantly increases the resistance of perovskite crystals to external stimuli. By incorporating MOFs into the design, the robustness of perovskite-based materials can be significantly improved^13^. During the solution processing of perovskite thin films, MOFs can act as mesoporous scaffolds by allowing perovskite nanocrystals to develop within their cavities due to their porous and flexible nature. The increased surface area of MOFs enables improved interfacial interaction between perovskite grains and the electron transport material (ETM), much like the mesoporous scaffolds of Al_2_O_3_ or TiO_2_ used in previous investigations^14,15^. This increased contact promotes efficient electron extraction and minimizes defect-induced interfacial charge recombination, thereby leading to improved PV performance. The MOF material, characterized by its porous structure, can act as a scaffold, facilitating the growth of the perovskite layer. Furthermore, the organic linkers present in MOF materials form coordination bonds with Pb^2+^, I^−^, and other functional groups within the perovskite structure, thereby influencing its crystallization process^14–16^. This interaction between the MOF and perovskite components plays a significant role in shaping the crystalline properties of the perovskite layer. For example, the insertion of a thin interlayer of ZIF-9 between compact TiO_2_ and perovskite layers can create additional channels for charge transport, resulting in an enhancement of the open-circuit voltage (Voc) in PSCs from 1.12 to 1.23 V^17^. In a study, we investigated the use of MOFs as interlayers to improve the performance and stability of HTL-free PSCs. Two types of MOFs, Cu-BTC and Co-BTC, were synthesized and spin-coated on TiO_2_ substrates. We characterized the morphology and size of the MOFs using SEM and analyzed the crystallinity and residual PbI_2_ of the perovskite films using XRD. The results showed that the Co-BTC MOF-based PSCs exhibited the highest PCE of 10.4% and the best stability, retaining 82% of their initial PCE after 264 h of storage in ambient air. The improved performance and stability were attributed to the enhanced crystallinity and reduced residual PbI_2_ of the perovskite film after Co-BTC MOF modification. Besides minimizing interfacial voltage losses, the uniform distribution of MOF crystals on the substrate plays a crucial role in controlling the crystallization kinetics and promoting the growth of larger perovskite grains in thin films, reaching the micrometer scale. This effect is attributed to the chemical coordination between the organic ligands present in MOFs and the perovskite precursors. The presence of MOFs facilitates favorable conditions for the formation of well-defined perovskite structures, leading to improved crystallinity and grain size in the resulting films. The MOFs selected for this study, Cu-BTC and Co-BTC, exhibit unique properties that make them stand out from other MOFs in the application of perovskite solar cells. Cu-BTC is renowned for its easy synthesis, excellent thermal stability, and good moisture resistance^18^. These characteristics are crucial for their integration into PSCs, where thermal and environmental stability are essential for long-term operation. Furthermore, Cu-BTC’s high porosity and surface area facilitate enhanced light absorption and provide ample sites for effective charge transfer, which are vital for the improved performance of PSCs^19^. The good pore volume and regeneration ability of Cu-BTC also contribute to its suitability as an interlayer in PSCs, promoting better crystallinity and reducing residual PbI_2_ in the perovskite film^20^. On the other hand, CoBTC is distinguished by its electrocatalytic properties, which are beneficial for the charge extraction process in PSCs^21^. The modification of Co-BTC with active groups has been shown to significantly enhance its efficiency in catalytic applications, suggesting that similar modifications could improve the performance of PSCs^22^. The choice of these MOFs over alternatives is further justified by the general advantages of MOFs in PSCs, such as enhanced charge-extraction efficiency, inhibition of charge recombination, and improved layer quality, all of which contribute to increased device stability. The tunable porous nanostructures and desirable chemical absorption characteristics of MOFs like Cu-BTC and Co-BTC play a beneficial role in constructing high-performance and eco-friendly PSCs^23^.

## Experimental

### Materials and methods

The chemicals used in this study are listed below, along with the companies that made them. All of the ingredients were utilized without further purification: We bought high-purity Cu(NO_3_)_2_∙3(H_2_O), CoCl_2_∙6(H_2_O), zinc powder (Zn), and 1,3,5-benzene tricarboxylate from Sigma Aldrich. Merck Chem. Co. (Germany) provided the following products: aniline, chlorobenzene, N,N-dimethylformamide (DMF, 99.9%), hydrochloric acid (HCl, 37%), dimethyl sulfoxide (DMSO, 99.9%), isopropanol (IPA, 99.7%), and anhydrous ethanol (99.99%). Sunlab Co. (IRAN) provided the following products: lead (II) iodide (PbI_2_, 99.99%), TiO_2_ paste (crystalline phase: anatase, particle size: 30 nm), carbon paste, methylammonium iodide (MAI, 99%), and fluorine tin oxide glass (FTO with 10–15 Ω/cm^2^).

For the structural study of these samples, wide-angle X-ray diffraction of the FTO/TiO_2_/perovskite and FTO/TiO_2_/MOFs/perovskite was recorded using XRD-Phillips X̕pert PRO with monochromatized Cu-Kα radiation (λ = 1.5178 Å). To ascertain the synthesis of MOFs, Tensor 27 spectrometer (Bruker, Saarbrucken, Germany) was used for Fourier transform infrared (FT-IR) spectroscopy. Using a field emission scanning electron microscope (FE-SEM Sigma, Zeiss) equipped with a gold coating, the morphological characteristics of MOFs and the surface morphological characteristics of perovskite films spin-coated on TiO_2_ and MOFs were recorded. Using a SPECORD 210 (Analytic Jena, Germany), the UV–Vis spectra of MOFs distributed in DMF under ultrasonic action were captured. The photocurrent density–voltage (J–V) performance of the PSCs was measured using an Auto Adjustable Solar Simulator (Karmana Photonics, Iran, with an AM 1.5, 100 mW/cm^2^) and a Solmetric I-V Curve Tracer (NanoSAT Co., Iran). The light's brightness was adjusted via a normative silicon cell. A 0.04 cm^2^ active area was produced by hiding the solar cells behind an aperture. All of the solar cells’ measurements were conducted in the ambient temperature and without any encapsulation.

#### Synthesis of [Co_3_(BTC)_2_(H_2_O)_n_]_n_ MOF

2.25 mmol equivalent to 0.54 g of cobalt (II) nitrate dissolved in 7.5 ml of deionized water and added to the ligand 1,3,5-benzene tricarboxylate (1.25 mmol, 0.262 g) dissolved in 7.5 ml of ethanol drop by drop in the ultrasonic bath, then we add chloroform drop by drop to the pink solution until a precipitate forms, and it is subjected to ultrasonic waves for 30 min, then the precipitate is filtered and washed with deionized water. We wash it several times and then dry it. (Color: white, melting point: 379 °C).

#### Synthesis of [Cu_3_(BTC)_2_(H_2_O)_3_]_n_ MOF

2.25 mmol is equivalent to 0.543 g of copper (II) nitrate dissolved in 7.5 ml of deionized water and added to the ligand 1,3,5-benzene tricarboxylate (1.25 mmol, 0.262 g) dissolved in 7.5 ml of ethanol drop by drop in the ultrasonic bath, and it is subjected to ultrasonic waves for 30 min in two stages, then the sediment is filtered and dried. (Color: blue, melting point: 387 °C).

### Perovskite solar cells fabrication

Zinc powder and an aqueous solution containing 2.0 M HCl were used to etch the FTO glass to obtain the necessary pattern before the solar cells were assembled. Then, utilizing ultrasonic cleaning, it was cleaned with deionized water, isopropanol, acetone, and anhydrous ethanol. To create the FTO/c-TiO_2_, a titanium diisopropoxide bis (acetylacetonate) solution in anhydrous ethanol (1:10 (v/v)) was spun onto the FTO glass for 45 s at 4500 rpm, and it was then heated for 30 min at 500 °C. Subsequently, FTO/c-TiO_2_/m-TiO_2_ electron transport layer (ETL) was produced by spin-coating TiO_2_ paste that had been diluted 1:5 in anhydrous ethanol, spun at 4500 rpm for 30 s, and annealed for 30 min at 500 °C. FTO/TiO_2_/MOF (Cu-BTC and Co-BTC) was made by spin-coating solutions of Cu-BTC MOF and Co-BTC MOF in anhydrous ethanol for 30 s at 4500 rpm. To eliminate the H_2_O between the molecules in the film, annealing was done for 30 min at 110 °C in the air. There are two processes involved in this process to prepare the active layer. The first stage involved spin-coating 461 mg of PbI_2_ in 1.0 ml of DMF and DMSO (in a 9:1 (v/v) ratio) on the ETL substrate for 20 s at 4000 rpm. Next, the PbI_2_ film was annealed for two minutes at 70 °C. The cells were submerged in a 30 mg/ml solution of MAI-IPA for 5 min in the second stage. After that, the substrate with the perovskite layer was heated for 15 min to 80 °C. Lastly, commercial carbon paste was used to deposit the carbon back-electrode onto the perovskite layer using the Doctor-Blade technique. After that, the entire apparatus was cured for 30 min at 100 °C.

## Results and discussion

### Characterization of MOFs

#### FT-IR analysis

**Figure 1:**
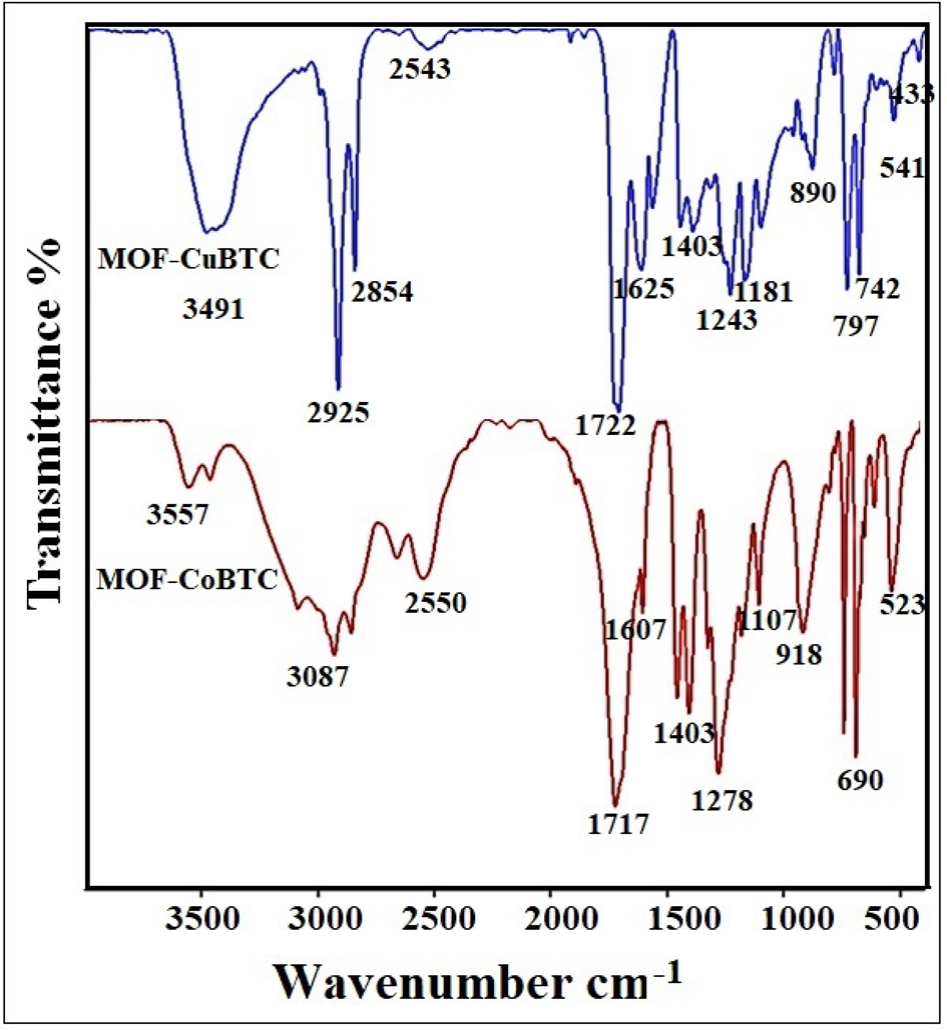
FT-IR spectra for Cu-BTC MOF and Co-BTC MOF.

FT-IR spectroscopies were used to describe the chemical structure of the MOFs (Cu-BTC and Co-BTC), which is depicted in Fig. 1. The results show that the C–H modes are assigned to comparatively mild absorption bands at 2800–3100/cm, while the large peak of O–H occurs at 3200–3500/cm. Strong bands corresponding to asymmetric and symmetric vibrational modes of C=O bonds are found in the 1600–1700/cm and 1300–1400/cm regions, respectively. Additional bands between 900 and 1200/cm are associated with the C–X (X: C, O) bond vibration. Additionally, a few bands observed at 400–600/cm match the Co–O and Cu–O interactions^24–28^.

**Figure 2:**
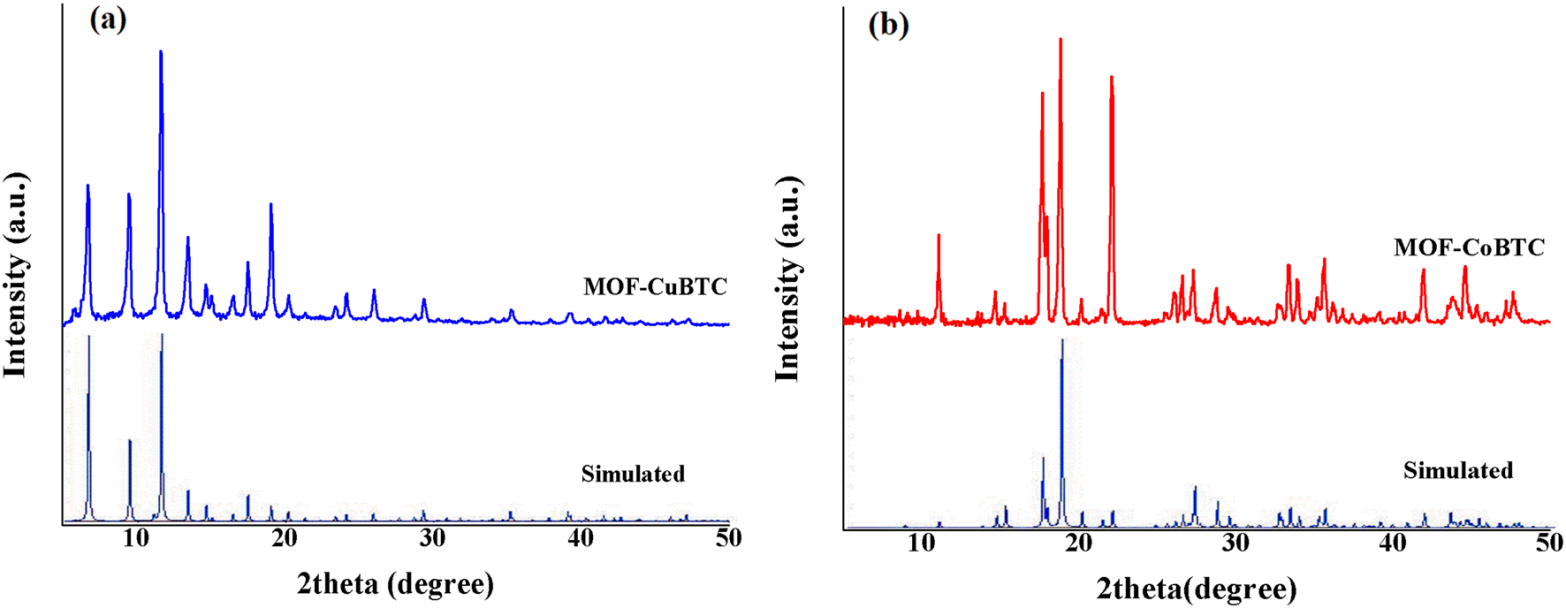
XRD patterns for (**a**) Cu-BTC MOF and (**b**) Co-BTC MOF.

To confirm the crystalline structure of the hierarchically porous metal-organic frameworks (MOFs) obtained, the powder X-ray diffraction (XRD) patterns of the synthesized MOF-Cu-BTC and MOF-Co-BTC samples were compared to the simulated patterns of Cu-BTC and Co-BTC (Cu–BTC for CCDC-112954 and CCDC: 921721 for Co-BTC)^28,29^. As depicted in Fig. 2a and b, the XRD patterns of MOF-Cu-BTC and MOF-Co-BTC exhibited sharp diffraction peaks that closely matched those of the simulated patterns. No additional peaks corresponding to impurities were observed, confirming the high purity of the synthesized products.

#### SEM analysis

**Figure 3:**
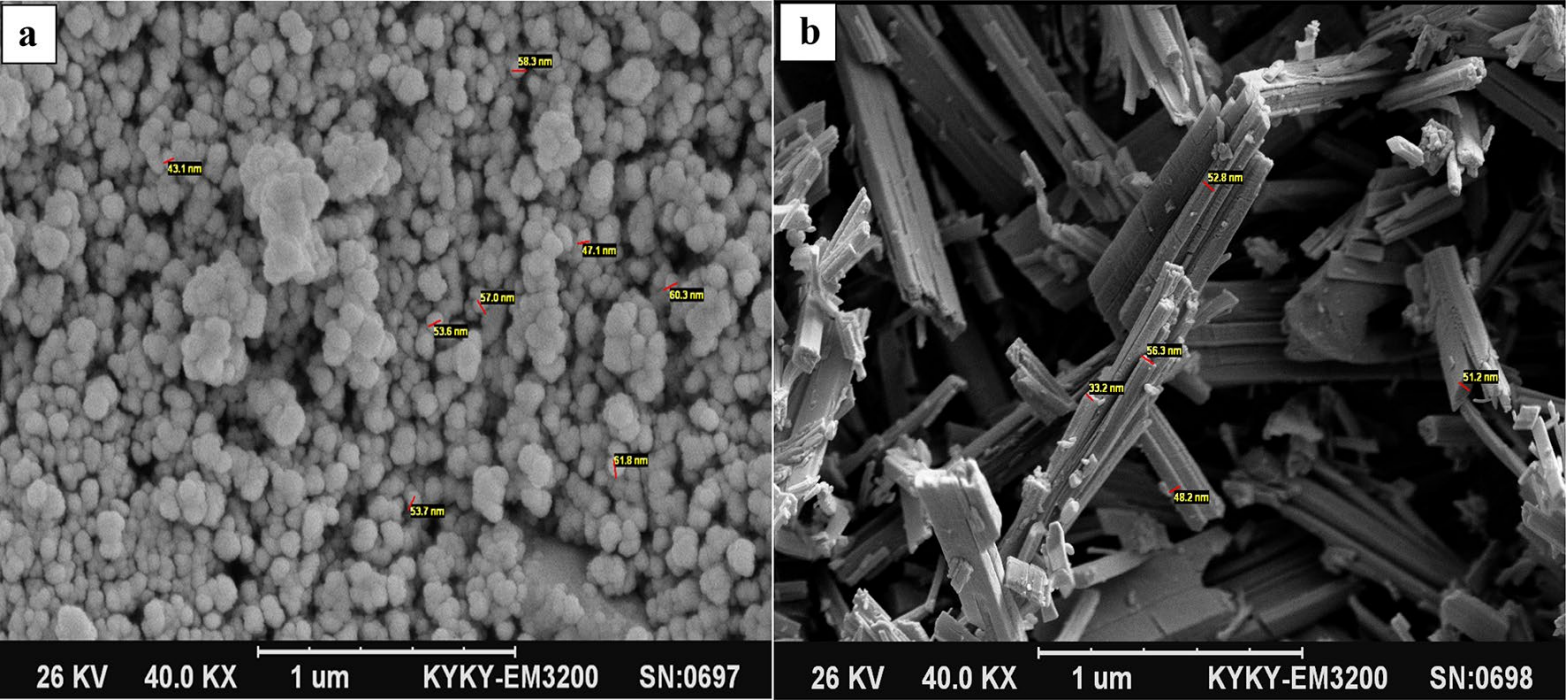
SEM images of (**a**) Cu-BTC MOF and (**b**) Co-BTC MOF.

The morphology and size of these metal-organic frameworks can be seen using the SEM images in Fig. 3a and b. The morphology of the MOF-CuBTC is spherical. Also, the particle size for this metal–organic framework is estimated between 43 and 60 nm. The SEM image shows that these nano-sphericals have a uniform dispersion.

The morphology of Co-BTC MOF is nano-rods that are placed together. Also, the diameter of the rods for this metal-organic framework is estimated between 33 and 56 nm.

#### Optical absorption spectroscopies

By using absorption spectra, the bandgaps of MOF (Cu-BTC and Co-BTC) were calculated. The UV–Vis spectroscopies provide the absorption data. For the absorption analysis, we made a DMSO solution (0.001 mM, 20 ml) and recorded room temperature spectra. Additionally, absorption bands are stretched from 200 to 800 nm wavelengths, and Tauc plots and optical absorption data were used to determine the matching wavelengths to the bandgaps^30^.

##### UV–visible analysis

**Figure 4:**
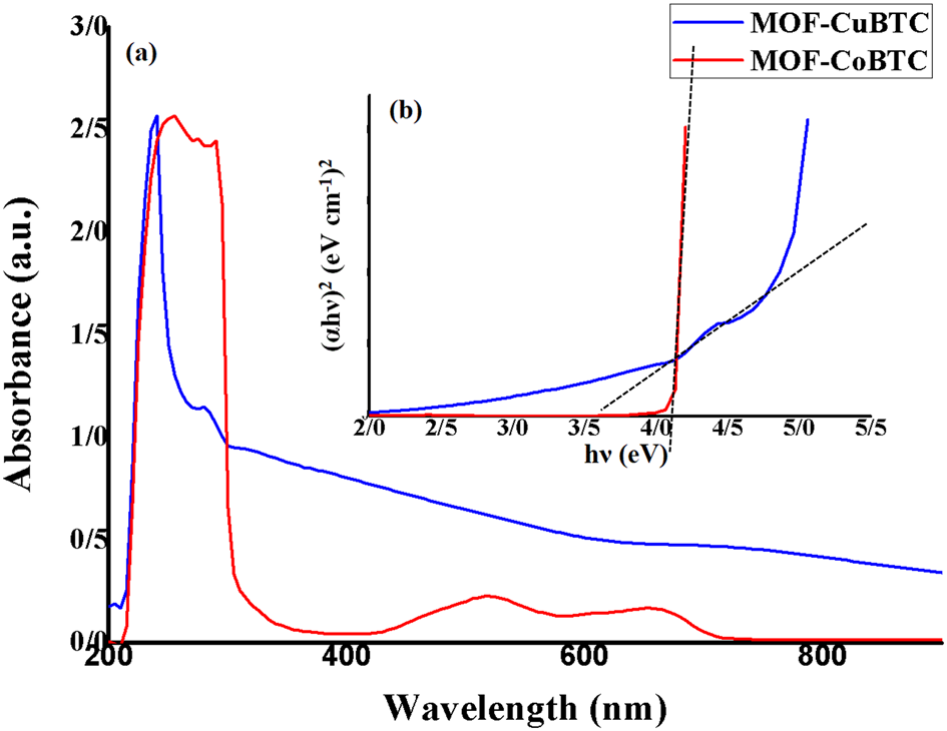
(**a**) UV − Vis absorption spectra of Cu-BTC MOF (blue) and Co-BTC MOF (red) and (**b**) Tauc plots showing indirect band gap values of Cu-BTC MOF (blue) and Co-BTC MOF (red).

The absorption spectra of MOFs (Cu-BTC and Co-BTC) are displayed in Fig. 4a. The π → π *ligand internal electron transfer is responsible for the absorption peak at 240 nm in the Cu-BTC UV–Vis absorption spectrum. An electron transition absorption peak known as metal-to-ligand charge transfer (MLCT) is observed at 280 nm. Additionally, the octahedral structure (Oh) of Cu(II) in Cu-BTC is assigned to the peak at 685 nm, which is associated to d → d electron transitions of Cu(II) ions^27^; ^2^Eg → ^2^T_2_g.

The Co-BTC UV–Vis absorption spectrum shows a peak at 245 nm, which is associated to electron transition π → π*, and another peak at 285 nm, which is associated with LMCT (O → Co and, N → Co). Also, some peaks at 515 and 655 nm are attributed to d → d electron transitions from the excited-state ^4^T_1_g (F) to the ^4^A_2_g and the ^4^T_1_g(P) due to Co (II)(d^7^) octahedral geometry in MOF-Co-BTC structure^31^.

An analysis of the absorption coefficient's dependence on photon energy in the high absorption area was one step toward obtaining comprehensive information regarding the energy gap for the samples. Using Tauc’s relation $\left( {\alpha {\text{h}}\nu } \right)^{{\frac{1}{{\text{r}}}}} = {\text{A}}\left[ {{\text{h}}\nu - E_{g} } \right]$, where A is a constant, ν is the frequency, h is the Planck constant, α is the light absorption index, and Eg is the semiconductor’s band gap, the optical band gap energy (Eg) is calculated. Depending on optical absorption, r takes on different values. For instance, the direct allowed transition is represented by 1/2, while the indirectly allowed transition is represented by 2.

To obtain complete information about the energy gap for the samples, an examination of the dependency of the absorption coefficient on photon energy in the high absorption area was one. The optical band gaps (Eg) are evaluated based on Tauc’s relation $\left( {\alpha {\text{h}}\nu } \right)^{{\frac{1}{{\text{r}}}}} = {\text{A}}\left[ {{\text{h}}\nu - E_{g} } \right],$ where A is a constant, $\nu$ is the frequency, h is the Planck constant, *α* is the light absorption index, and *E*_*g*_ is the band gap of the semiconductor, respectively. The graph between (αhv)^2^ and (hv) in eV is shown in Fig. 4b to help determine the energy bandgap. According to research, the band gaps of CuBTC (a) and CoBTC (b) are 3.52 and 4.2 eV, respectively^29^.

The optical characteristics of the Cu-BTC and Co-BTC MOFs make them well-suited for use in PSCs. Their ability to convert high-energy photons to lower energies through the down-conversion process could potentially benefit the perovskite film layer^32–35^. Additionally, their strong absorption in the ultraviolet (UV) region allows them to act as a UV filter, which is advantageous since UV radiation has been reported to be detrimental to perovskite materials.

### Perovskite films analysis

The presence of metal-organic frameworks (MOFs) in the perovskite film fabrication process could affect the morphology and performance of the resulting perovskite film.

By incorporating the MOF-induced rapid nucleation method into the fabrication process, the nucleation and crystallization of perovskite can be accelerated, leading to the formation of more small domains. These small domains can effectively fill the pinholes and voids and the result of this filing in perovskite film improved morphology and produced larger grains, and fewer grain boundaries. The method offers better control of the crystallinity and surface coverage of the perovskite film, ultimately enhancing its overall quality and performance. The combined effects of enhanced crystallinity, improved morphology, and reduced defects in the perovskite film can significantly impact its performance. These factors can enhance charge carrier mobility, reduce trap states, and improve the overall efficiency and stability of perovskite solar cells. It's worth noting that the specific influence of MOFs on perovskite film morphology and performance may vary depending on the choice of MOFs, their properties, and the fabrication parameters employed. Therefore, careful selection and optimization of the MOF-induced nucleation method are crucial to achieving the desired improvements in the perovskite film’s morphology and performance.

#### XRD analysis

**Figure 5:**
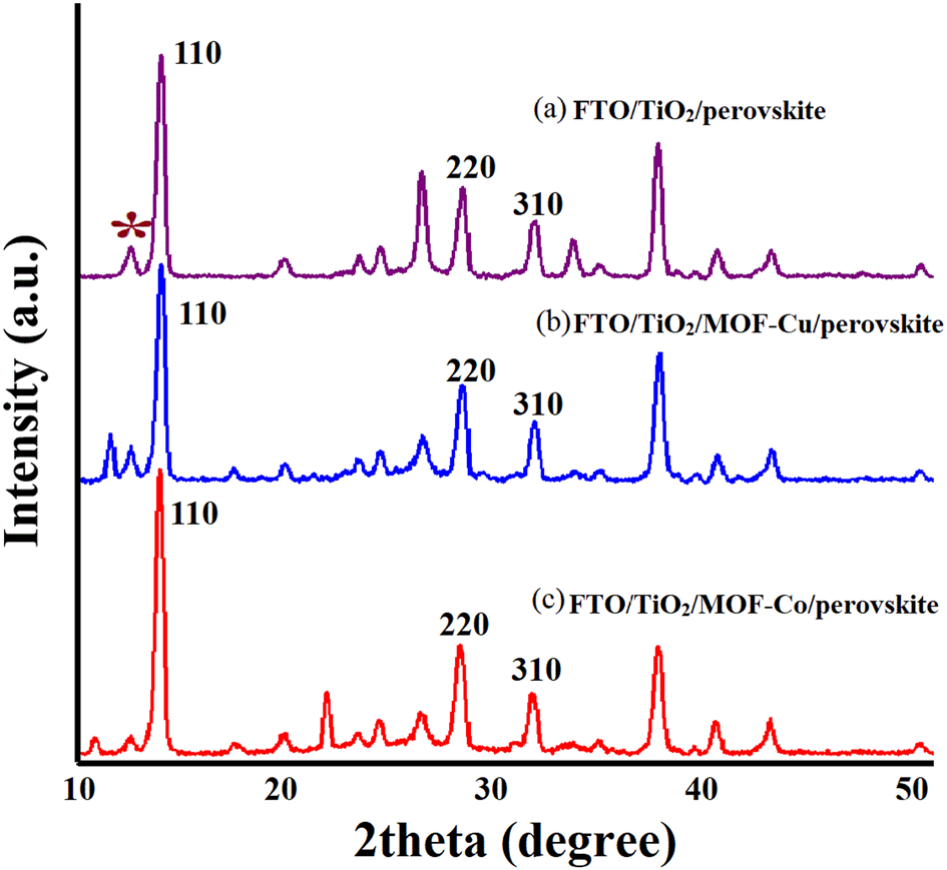
XRD patterns of different (**a**) FTO/TiO_2_/MAPbI_3_, (**b**) FTO/TiO_2_/Cu-BTC MOF/MAPbI_3_, and (**c**) FTO/TiO_2_/Co-BTC MOF/MAPbI_3_ structures.

The X-ray diffraction (XRD) patterns of perovskite films on pristine and MOF (Cu-BTC and Co-BTC)-modified TiO_2_ substrates are compared in Fig. 5. The (110), (220), and (310) planes of the perovskite layer correspond to the distinctive diffraction peaks at 14.04°, 28.46°, and 31.90°^34^. The presence of a peak around 10° in the XRD patterns of the MOF-modified perovskite layers can be attributed to the characteristic diffraction peaks of the MOF materials incorporated into the perovskite films. The MOF materials, such as Cu-BTC and Co-BTC, have distinct XRD patterns (Fig. 2) with characteristic peaks that can be observed in the modified perovskite films. By analyzing the XRD pattern of the perovskite layer, we observed changes in the Co-BTC and Cu-BTC-modified perovskite films compared to the unmodified film, FTO/TiO_2_/perovskite. In the Co-BTC and Cu-BTC-modified samples, the peak intensities of the (110) peak are enhanced, indicating an improvement in the crystallinity of the perovskite film. Additionally, the full width at half maximum (FWHM) of the peak is reduced, suggesting a narrower peak and a more well-defined crystal structure, which further supports the improved crystallinity.

During the annealing process of perovskite films, a PbI_2_ cubic structure can be formed, leading to a detectable signal's presence at 12.7° in the XRD pattern^35^. The presence of a PbI_2_ peak indicates that there is still residual PbI_2_ in the film. However, in the Co-BTC and Cu-BTC-modified sample, the peak intensities of the (PbI_2_) peak are reduced compared to the pristine sample. This reduction implies a decrease in the amount of residual PbI_2_ in the perovskite film after MOF modification.

The improved crystallinity of the perovskite film and the reduction of residual PbI_2_ after MOF modification are expected to have a positive impact on the current density in the perovskite solar cell. The enhanced crystallinity can contribute to better charge transport properties, while the reduction of residual PbI_2_ can minimize recombination, leading to improved device performance. The end groups of MOFs (Cu-BTC and Co-BTC) can create robust hydrogen bonds with halide anions in the perovskite material. This interaction potentially leads to a favored formation of nuclei at the MOF sites and promotes the development of polycrystalline perovskite thin films with aligned facet orientations^32^. Importantly, the strong chemical bonding between the MOF framework and perovskite layers can additionally improve the adhesion of perovskite thin films to their supporting substrates.

#### FE-SEM analysis

The perovskite layer is where the majority of the photoelectric conversion, carrier dissociation, and transfer processes occur in perovskite solar cells. Improving the morphology of perovskite films is crucial for their stability and performance. One common issue with CH_3_NH_3_PbI_3_ films is their susceptibility to deterioration in air-conditioned environments, primarily due to moisture penetration through grain boundaries. Therefore, enhancing the perovskite morphology is an effective approach to reducing the instability of CH_3_NH_3_PbI_3_ films^35^.

There are two main reasons why a large perovskite crystal size is preferred. Firstly, grain boundaries can act as charge recombination centers, negatively impacting the performance of the solar cell. By increasing the crystal size, the density of grain boundaries per volume decreases, reducing charge recombination and improving the open-circuit voltage (Voc) and fill factor (FF) of the solar cell^36,37^. Secondly, larger perovskite crystals facilitate the movement of photoinduced charges over longer distances during the charge transfer process, leading to enhanced device performance^38^.

**Figure 6:**
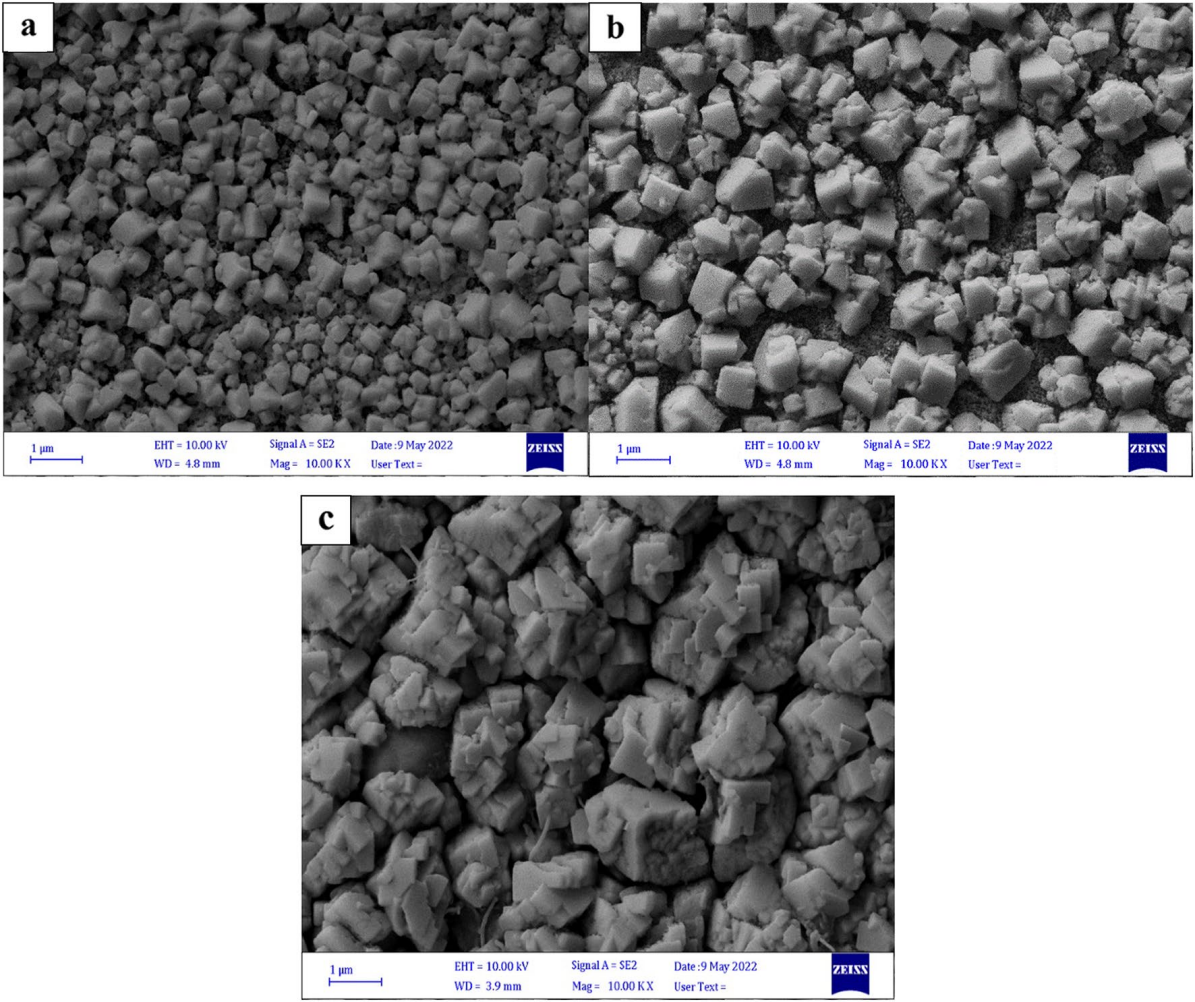
Top-view SEM images of the MAPbI_3_ perovskite films deposited on (**a**) FTO/TiO_2_, (**b**) FTO/TiO_2_/Cu-BTC MOF, and (**c**) FTO/TiO_2_/Co-BTC-MOF.

To investigate the influence of perovskite film growth on different surfaces, scanning electron microscopy (SEM) analysis was conducted. Figure 6a–c illustrates the SEM images obtained from the perovskite films grown on the surfaces. It is evident from the observations that the perovskite formed on the titanium dioxide (TiO_2_) ETL with the MOF interlayers exhibits larger grain sizes compared to those grown on the pristine TiO_2_ surface. This indicates that the MOF film has a positive impact on the growth and size of perovskite grains while reducing the grain boundaries and defects of the perovskite film. The literature has shown that this extra MOF scaffold may facilitate the nucleation of perovskites during the film's evolution. In the meantime, the MOF structure’s polar groups may momentarily coordinate with Pb^2+^ to adjust the rate of crystallization, increasing the prepared film’s grain size^39–42^.

It is important to note that water has a lower boiling point and higher vapor pressure than either dimethylformamide (DMF) or dimethyl sulfoxide (DMSO). The presence of moisture attracted by the Co-BTC and Cu-BTC MOFs slows down the solvent evaporation rate from the perovskite film. As a result, larger grains are formed, which is beneficial for enhancing the performance and stability of the device^41^.

Micro-porous Co-BTC and Cu-BTC nanocrystals are used as a conventional scaffold so that perovskite crystals occur inside. Therefore, this traditional scaffold provides an orderly arrangement of perovskite microcrystals during the initial stage of crystallization. As shown in Fig. 8, it can be observed that the Co-BTC MOF-based PSCs’ perform best among all solar cells, with an average PCE of 10.4%. The perovskite layer on the Co-BTC MOF surface exhibits a larger size and fewer grain boundaries, likely due to the rod-shaped morphology of this particular MOF. However, the perovskite crystal on TiO_2_ with a Co-BTC MOF interlayer appears more homogeneous than pure TiO_2_. This indicates that Co-BTC MOF can optimize the perovskite morphology compared to pure TiO_2_ and TiO_2_ with a Cu-BTC MOF interlayer. Achieving full coverage and a uniform perovskite layer is advantageous for light absorption and charge transport. A homogeneous perovskite layer with good surface coverage is ideal for high-performance solar cells, as any pinholes could create direct contact between the ETL and the HTL, resulting in a shunting channel.

**Figure 7:**
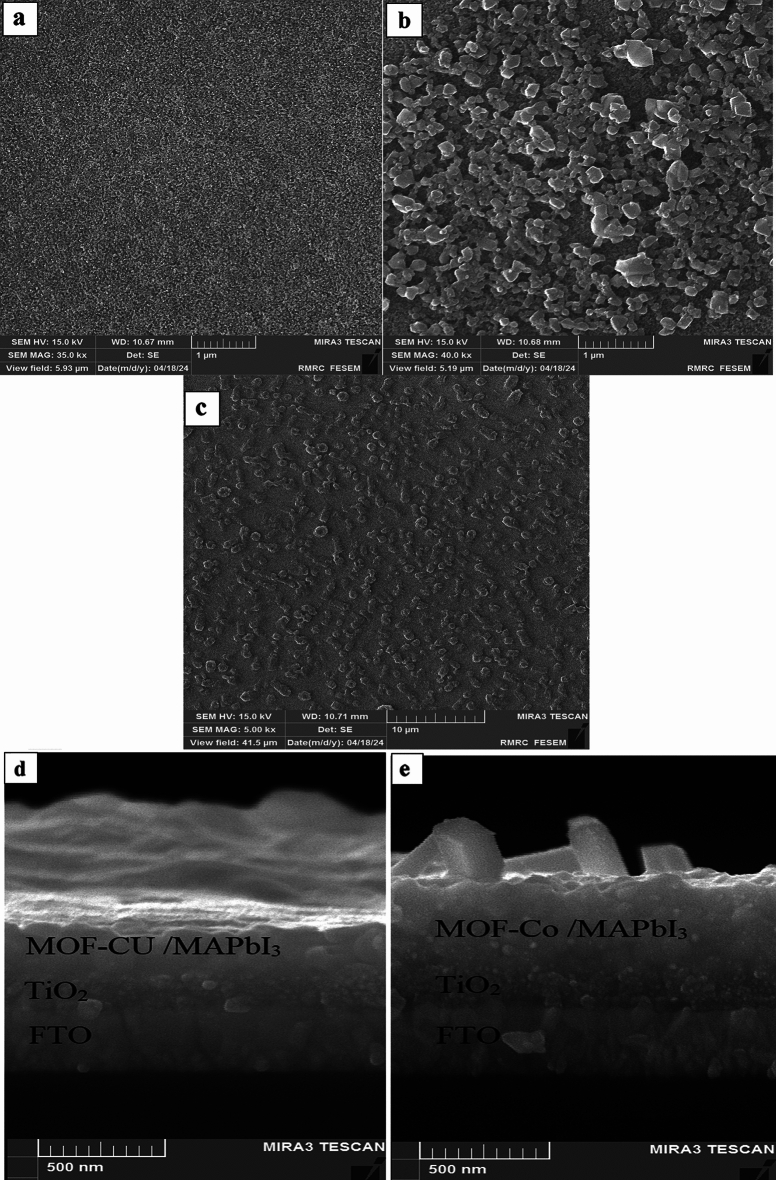
The top view SEM of (**a**) TiO_2_, (**b**) Cu-BTC MOF on TiO_2_, (**c**) Co-BTC MOF on TiO_2_. The cross-section of the device having FTO/TiO_2_/MOF/MAPbI_3_, (**d**) Cu-BTC MOF, and (**e**) Co-BTC MOF.

Figure 7 shows the top view SEM images of TiO_2_, Cu-BTC MOF, and Co-BTC MOF on TiO_2_. Figure 7a displays a relatively smooth and uniform surface, which is the titanium dioxide (TiO_2_) layer. Figure 7b and c depict SEM images illustrating the different surface morphologies resulting from the deposition of MOF materials (Cu-BTC and Co-BTC) on a TiO_2_ base layer. The MOF coatings introduce a more porous surface texture compared to the bare TiO_2_. This porosity can be attributed to the crystalline or porous nature of these MOF materials.

In order to investigate the impact of film thickness on the photovoltaic performance of perovskite solar cells, cross-sectional SEM images of devices featuring the layer structure FTO/TiO_2_/MOF/MAPbI_3_ are displayed in Fig. 3d and e.

#### UV–Visible and Photoluminescence (PL) analysis

**Figure 8:**
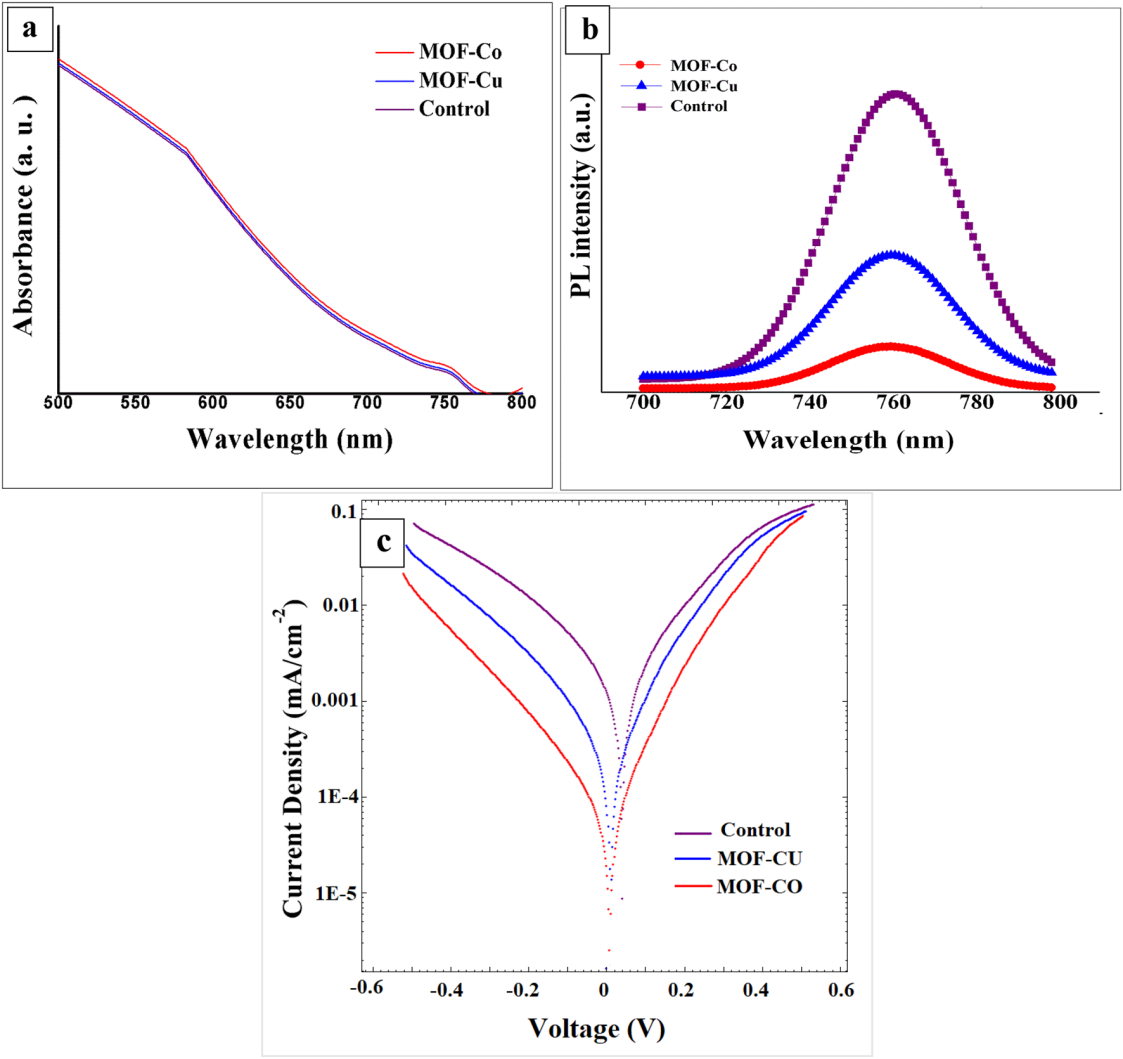
(**a**) Absorbance spectrum, (**b**) PL of different FTO/TiO_2_/MAPbI_3_, FTO/TiO_2_/Cu-BTC MOF/MAPbI_3_, and FTO/TiO_2_/Co-BTC MOF/MAPbI_3_ structures, and (**c**) the Tafel response of different net and modified ETLs.

The enhanced crystallinity of the perovskite layer fabricated on TiO_2_ modified with MOFs contributes to its increased absorption power. This conclusion is supported by a comparison of the UV–visible absorption spectra of the fabricated perovskite layers using different configurations, as depicted in Fig. 8a. The spectra show minimal changes, suggesting that the presence of the MOF layer has a negligible impact on the perovskite layer's light absorption capability, while slightly enhancing the absorption intensity. These results align with the findings from SEM and XRD experiments, indicating that the perovskite film can absorb more photons, generating a higher electron count and consequently resulting in increased current output.

Moreover, the absorption spectrum of the MAPbI_3_ perovskite layer exhibits a prominent peak at around 760 nm. This peak confirms that the perovskite layer has been successfully formed and possesses the ability to absorb light within the visible range of the electromagnetic spectrum^42^.

The steady-state photoluminescence (PL) spectra of perovskite films fabricated on various layers were investigated as presented in Fig. 8b. The thin films consisting of FTO/TiO_2_/MOFs/MAPbI_3_ exhibited a remarkably narrow emission band centered at 763 nm, as depicted in Fig. 8b. This emission band can be attributed to the excitonic recombination process commonly observed in TiO_2_. Interestingly, when the MOF layer was added to the TiO_2_ ETL, there was no discernible change in the position of this emission band. However, the incorporation of the MOF layer on the TiO_2_ ETL resulted in a more pronounced quenching effect on the photoluminescence intensity of the perovskite films compared to the films with TiO_2_ ETL. This enhanced quenching effect signifies a more efficient extraction of electrons or holes across the interfaces between the perovskite layer and the ETL^43,44^. The most photoluminescence quenching occurred when the MAPbI_3_ perovskite film was deposited on the Co-BTC layer. This observation strongly suggests that charge carriers generated within the perovskite absorbers can migrate more efficiently through the Co-BTC. The improvement in interfacial compatibility and charge extraction efficiency can be ascribed to the 3D porous scaffold of MOF, which facilitates the filling of perovskite precursors and the creation of perovskite nanocrystals^39,45,46^. Consequently, this improved charge carrier mobility facilitates their faster extraction and transport across the perovskite-ETL interface, which holds promise for enhancing the overall performance of perovskite-based devices.

In the meantime, as Fig. 8c shows, the nonradiative recombination can be deduced from the reverse saturation current density in the absence of light. The Co-BTC MOF-modified device exhibited reduced dark current, which proved that the device had less energy loss during operation. As in the case of the PSCs with the Co-BTC MOF layer, lesser nonradiative recombination is correlated with a lower dark current. As a result, the Co-BTC MOF-based PSCs’ V oc dramatically increased.

#### Photovoltaic properties of PSCs

To investigate the advantages of MOF layers, we conducted an experiment to fabricate PSCs with and without the addition of MOF layers, specifically CO-BTC and Cu-BTC. The device structure used was FTO/TiO_2_/MOF/perovskite/carbon.

**Figure 9:**
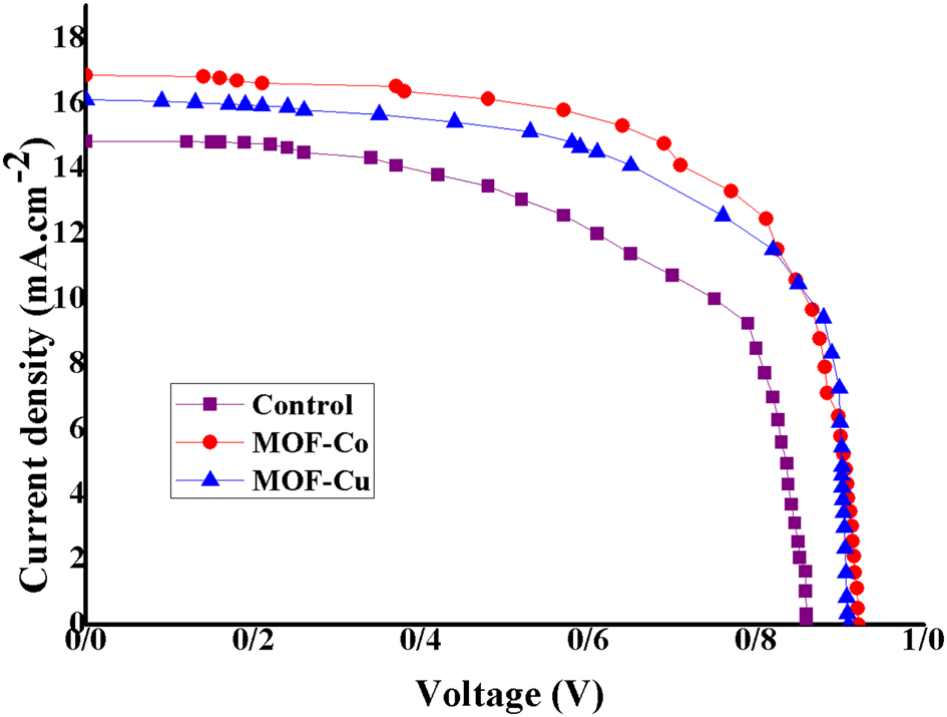
The J-V tests of the PSCs based on FTO/TiO_2_/MAPbI_3_/C, FTO/TiO_2_/Cu-BTC MOF/MAPbI_3_/C, and FTO/TiO_2_/Co-BTC MOF/MAPbI_3_/C.

We evaluated the photovoltaic characteristics of these champion PSCs by measuring the J-V (current–voltage) curves under AM 1.5G illumination conditions. The results of our measurements are summarized in Table 1. Upon comparing the performance of PSCs with and without the MOF layers (Fig. 9), we observed that the incorporation of MOF layers, particularly Co-BTC, resulted in an improved PCE in the devices. Notably, we achieved the highest PCE performance of 10.4% when the device was fabricated with Co-BTC. These findings underscore the beneficial influence of Co-BTC in the context of perovskite solar cells. The presence of the Co-BTC layer on the TiO_2_ ETL positively impacted the device performance, leading to a higher PCE. This improvement can be attributed to several factors, including enhanced charge carrier transport, reduced recombination losses, and improved overall device stability. The increased FF was attributed to the improved interfacial electrical contact as mentioned, while the improved Jsc was ascribed to the improved light-harvesting capability of the perovskite film owing to the promoted crystallization (Fig. 7a).

**Table 1:** Photovoltaic performance PSCs based on FTO/TiO_2_/MAPbI_3_/C, FTO/TiO_2_/Cu-BTC MOF/MAPbI_3_/C, and FTO/TiO_2_/Co-BTC MOF/MAPbI_3_/C.

Device name		J_SC_ (mA/cm^2^)	V_OC_ (V)	FF	ƞ (%)
Control	Average	14.53 ± 0.179	0.84 ± 0.01	0.57 ± 0.015	6.8 ± 0.24
MOF-Cu	Average	16.00 ± 0.142	0.90 ± 0.0035	0.62 ± 0.017	9.01 ± 0.378
MOF-Co	Average	16.36 ± 0.449	0.902 ± 0.027	0.64 ± 0.02	9.46 ± 0.612

**Figure 10:**
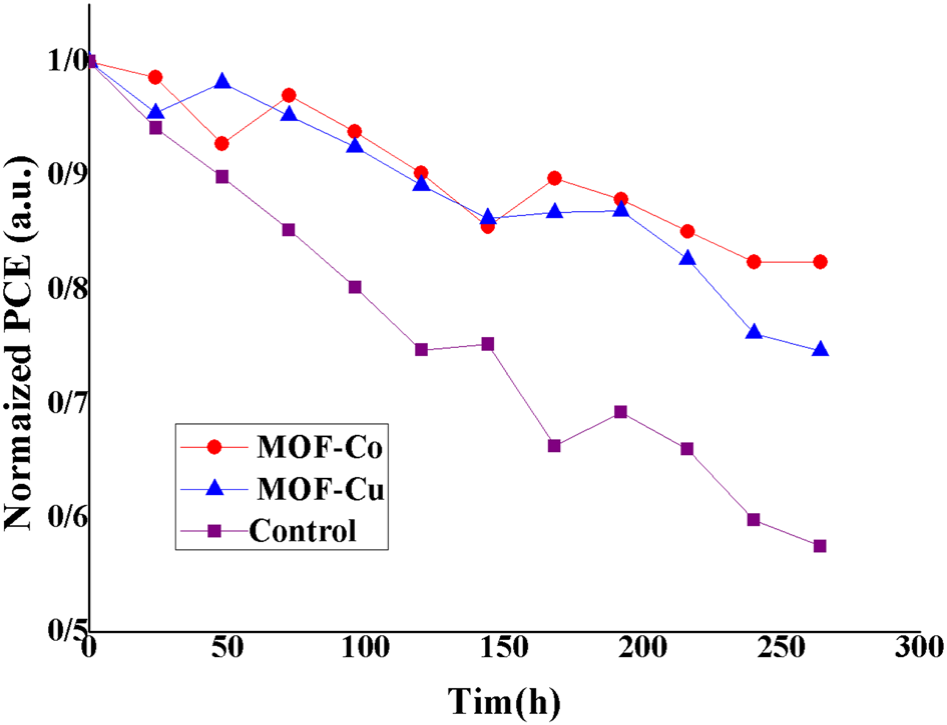
Stability test of perovskite solar cells for 264 h (All devices are placed in ambient air).

Perovskite solar cells' stability is a crucial factor that warrants careful consideration. Figure 10 presents the outcomes of our investigation on the stability of both optimized and unoptimized perovskite photovoltaic solar cells. To compare the efficiency of the optimized and non-optimized solar cells, we exposed them to ambient conditions (approximately 25 ℃ and 45% humidity) for approximately 264 h. The results indicate that the efficiency of the device optimized with a Co-BTC layer remained at 82% of its initial efficiency, while the efficiency of the unoptimized photovoltaic device dropped to approximately 57% of its initial efficiency. These measurements suggest that the photovoltaic solar cells optimized with Co-BTC and Cu-BTC layers exhibit greater stability compared to those with TiO_2_. The improved stability can be attributed to the inclusion of the MOF layer, which effectively reduces surface defects and minimizes electron–hole recombination between the perovskite layer and the electron transport layer. These factors contribute to slowing down the degradation of perovskite photovoltaic solar cells and ultimately enhancing their overall stability. In conclusion, the implementation of MOF (Cu-BTC and Co-BTC) interlayers has demonstrated significant potential for enhancing the long-term stability of PSCs against various external factors.

Metal-organic frameworks offer a unique porous structure and chemical functionality, making them suitable as interlayers to enhance the resilience of perovskite crystals against external stimuli. The high surface area of MOFs facilitates improved interfacial contact between perovskite grains and the electron transport material, leading to efficient electron extraction and reduced interfacial charge recombination. Additionally, the uniform distribution of MOF crystals on the substrate promotes the growth of larger perovskite grains, resulting in improved crystallinity and grain size of the thin films. The interaction between MOFs and perovskite components plays a significant role in shaping the crystalline properties of the perovskite layer.

## Conclusion

This research paper investigates the use of MOF nanostructures with different morphologies, spherical (Cu-BTC) and rod (Co-BTC) as interlayers in HTL-free PSCs to enhance their performance and stability. Two types of MOFs, Cu-BTC and Co-BTC, were synthesized and spin-coated on TiO_2_ substrates to form the PSCs. The results demonstrate that the MOF-CoBTC PSC exhibits the highest PCE of 10.4% and the best stability, retaining 82% of its initial PCE after 264 h of storage in ambient air. These improvements are attributed to the enhanced crystallinity and reduced residual PbI_2_ of the perovskite film after Co-BTC modification. Employing MOF interlayers, Co-BTC, enhanced the absorption of light by perovskite layers and, at the same time, alleviated the undesirable decomposition of perovskite caused by the penetration of UV light. When compared to conventional TiO_2_ mesoporous scaffold layers, the combination of TiO_2_ and MOFs provides a sturdier and more organized scaffold. The study highlights the potential of MOF-based interlayers as a promising alternative for HTL-free PSCs. Overall, this research investigating the impact of different MOF structures on the performance of PSCs could shed light on the relationship between MOF morphology and device efficiency and contribute to the ongoing efforts to enhance the efficiency and durability of perovskite solar cells for practical applications in photovoltaics. Moving forward, the potential of MOFs of enhancing perovskite solar cells invites a multitude of research opportunities. Future work could focus on diversifying MOF structures to fine-tune their interaction with perovskite layers, optimizing MOF synthesis for greater purity and uniformity, and investigating the interfaces for improved charge transfer. Additionally, extended stability assessments and the exploration of scalable fabrication methods are essential for transitioning to commercial viability. Lastly, a comprehensive environmental impact study of MOF-based PSCs will ensure their sustainability, marking a significant stride toward the practical application of photovoltaics.

## Data Availability

The authors declare that the data supporting the findings of this study are available within the paper. Should any raw data files be needed in another format they are available from the corresponding author upon reasonable request.
